# Insomnia symptoms and stress exposure interact in relation to Alzheimer’s disease biomarkers

**DOI:** 10.1007/s00415-026-14102-7

**Published:** 2026-09-03

**Authors:** Jasper Holleman, Ingemar Kåreholt, Manasa Shanta Näsholm, Charlotte Sørensen, Göran Hagman, Malin Aspö, Miia Kivipelto, Alina Solomon, Shireen Sindi

**Affiliations:** 1 Division of Clinical Geriatrics, Department of Neurobiology, Care Sciences, and Society, Karolinska Institutet, Karolinska Vägen 37 A - QA32, Solna, 171 64 Stockholm, Sweden; 2 School of Health and Welfare, Aging Research Network –Jönköping (ARN-J), Institute of Gerontology, Jönköping University, Jönköping, Sweden; 3 Aging Research Center, Theme Inflammation and Aging, Karolinska Institutet and Stockholm University, Stockholm, Sweden; 4 Cognitive Disorders Clinic, Karolinska University Hospital, Stockholm, Sweden; 5 Ageing Epidemiology Research Unit (AGE), School of Public Health, Faculty of Medicine, Imperial College London, London, UK; 6 Institute of Public Health and Clinical Nutrition, University of Eastern Finland, Kuopio, Finland; 7 Center for Aging Well, Yale School of Nursing, Yale University, West Haven, Connecticut USA; 8 Department of Neurology, Institute of Clinical Medicine, University of Eastern Finland, Kuopio, Finland

**Keywords:** Stress, Insomnia, Sleep, Alzheimer’s disease, Beta-Amyloid, Aging

## Abstract

**Background:**

Several modifiable factors affect Alzheimer’s disease (AD) risk. However, evidence on how these factors interact remains scarce, limiting our understanding of their role in AD development. This study aims to assess interactions between chronic stress exposure and insomnia in relation to AD biomarkers beta-amyloid (Aβ_42_), total tau, and phosphorylated tau.

**Methods:**

The present study included 124 memory clinic patients without dementia from the Cortisol and Stress in Alzheimer’s disease (Co-STAR) cohort study. Insomnia symptoms, stressful life events (SLEs) and current perceived stress were self-reported via questionnaires, while AD biomarkers were assessed from the cerebrospinal fluid (CSF). Cross-sectional interactions between stress and insomnia in relation to AD biomarkers were examined using linear regression models.

**Results:**

Insomnia and SLEs interacted in relation to Aβ_42_. Greater stressor exposure was associated with reduced CSF Aβ_42_ levels, reflecting greater brain amyloid accumulation, only in those with moderate-to-high insomnia scores. No interactions were found for total or phosphorylated tau.

**Conclusions:**

This study suggests that chronic stress and sleep disturbances exhibit an interactive relationship in their associations with AD pathology, and highlights the need for further research into interactive effects of multiple modifiable risk factors in the development of AD.

**Supplementary Information:**

The online version contains supplementary material available at https://doi.org/10.1007/s00415-026-14102-7.

## Introduction

Alzheimer’s disease (AD) poses a major public health challenge in aging societies [57]. Its characteristic pathological processes are commonly assessed using cerebrospinal fluid (CSF) biomarkers, such as decreased beta-amyloid (Aβ)_42_, reflecting Aβ accumulation, increased phosphorylated tau (P-Tau), reflecting tau accumulation, and increased total tau (T-Tau), reflecting neurodegeneration [10, 27]. Aβ and tau accumulation have been common targets of drug development efforts for AD [64]. However, prevention remains the most effective method of reducing the AD burden [35]. The 2024 Lancet Commission Report on dementia prevention estimated that up to 45% of dementia cases could be delayed or prevented by reducing 14 modifiable risk factors [35]. Beyond these established risk factors, several promising factors may offer additional preventive potential.

One such factor is disturbed sleep [35]. Sleep disturbances often present as insomnia in older adults, characterized by difficulty falling asleep, frequent nightly awakenings, and/or early morning awakenings [46]. Insomnia has been linked with dementia risk [3, 30, 31, 35] with Aβ and tau accumulation potentially mediating this association [22]. Sleep facilitates glymphatic system activity, one function of which is the clearance of proteins, such as Aβ and tau from the brain [5]. Insomnia, therefore, reduces glymphatic clearance [5], and has consequently been found to be associated with elevated CSF Aβ_42_ levels in middle-aged and older adults [17, 39, 63].

In older adults, insomnia is closely related to other modifiable factors that may themselves influence AD risk. However, research investigating potential interactive effects between multiple related risk factors in relation to AD pathology remains rare. Ignoring the interplay between closely related modifiable factors may yield an incomplete understanding of their role in the development of AD. Chronis stress, which has a close, bi-directional association with insomnia, may be one such factor [18, 31]. Like insomnia, chronic stress is thought to increase AD risk [21, 65], possibly through pathways involving Aβ and tau [2, 12, 32, 43]. Consequently, insomnia-related reductions in Aβ clearance and chronic stress-related increases in AD biomarker production and accumulation may interact to promote AD pathology beyond the individual effects of either risk factor.

The limited available evidence supports the potential existence of such interactive effects. For instance, one study in younger women found that stressor exposure was only associated with cognitive function among those with frequent nightly awakenings (a common aspect of insomnia) [19]. Additionally, animal research has found that reduced Aβ clearance resulting from physical inactivity, another modifiable risk factor, may weaken resilience against the effects of chronic stress exposure [62]. Since insomnia also reduces Aβ clearance [5], it may similarly weaken stress resilience. Research directly assessing these interactions is needed to gain a more complete understanding of chronic stress and insomnia as potential AD risk factors.

This study aims to assess interactions between self-reported stress exposure and insomnia symptoms in relation to CSF AD biomarkers Aβ_42_, T-Tau, and P-Tau181 in a sample of memory clinic patients. This population tends to be at elevated risk of high stress and sleep disturbances compared to cognitively healthy older adults [13, 30]. Stress and insomnia are hypothesized to interact such that the associations between greater stress exposure and reduced CSF Aβ_42_ levels as well as elevated T-Tau and P-Tau181 levels is strongest among individuals with more severe insomnia symptoms.

## Methods

### Study design and participants

This cross-sectional study is based on baseline data from the Cortisol and Stress in Alzheimer’s disease (Co-STAR) cohort study on memory clinic patients. The Co-STAR study was conducted at the Karolinska University Hospital memory clinic in Stockholm County, Sweden. Participants were recruited from patients visiting the clinic for the first time between 2014 and 2017, and underwent all standard clinical assessments, including lumbar punctures for the collection of CSF and neurocognitive testing. The cognitive test battery included examinations related to a variety of cognitive domains, including episodic memory (Rey Auditory Verbal Learning Test (delayed recall) [52], the Rey-Osterrieth Complex Figure (immediate recall) [42], the immediate recall of digit-symbol pairs from the Wechsler Adult Intelligence Scale (WAIS) Digit Symbol Substitution Test [58], and the Hagman test, developed at the Karolinska University Hospital to assess visual memory), working memory (WAIS Digit Span and WAIS Arithmetic), processing speed (WAIS Digit Symbol Substitution test), perceptual reasoning (WAIS Block Design and WAIS Matrices) [58]. Co-STAR participants were additionally requested to fill out a series of questionnaires related to stress exposure, sleep, and other lifestyle-related factors.

Following clinical routines, all patients received a diagnosis based on a consensus meeting. Dementia diagnoses were based on the International Classification of Diseases 10th Revision (ICD-10) [60], diagnoses of mild cognitive impairment (MCI) were based on the Winblad et al*.* criteria [59]. Individuals who were referred to the memory clinic due to perceived cognitive decline, but who did not perform below the norms on the neurocognitive tests used to assess the presence of dementia or MCI, were given a diagnosis of subjective cognitive impairment (SCI), in line with the criteria specified by Jessen et al., which include self-experienced persistent decline in cognitive capacity with normal age-, gender-, and education-adjusted performance on cognitive tests, without the presence of MCI, prodromal AD, or dementia [28]. The Co-STAR sample has been described in greater detail previously [24]. Due to the use of self-reported questionnaire data, 41 participants with a dementia diagnosis were excluded from the full Co-STAR sample of 188 participants, as was one individual for whom information regarding diagnosis was missing, and 22 without available data on insomnia and stress exposure, yielding a final sample of 124 participants.

### Insomnia and stress measures

#### Insomnia

Self-reported insomnia symptoms were assessed using three items from the validated Karolinska Sleep Questionnaire (KSQ) [41]. Based on the following phrasing: “Have you been bothered by the following complaints during the past three months”, the items of interest were: “difficulties falling asleep”, “repeated awakenings with difficulties falling asleep again”, and “premature awakenings”. Responses were recorded on a six-point Likert scale, ranging from ‘never’ (score of 0) to ‘always (5+ times a week)’ (score of 5) [41]. Responses to these three questions were summed to create one overall insomnia score, ranging from 0 to 15, with greater scores indicating more severe insomnia symptoms. While the KSQ covers multiple aspects of sleep quality and quantity, the present study focused solely on insomnia (a common sleep disturbance in older adults [46], which has previously been found to be associated with dementia risk [3], AD biomarkers [5], and chronic stress [31]) to reduce the number of required analyses.

#### Stress measures

Chronic stress exposure was assessed by means of two questionnaires: the 10-item Perceived Stress Scale (PSS) assessing current perceived stress [40], and a 16-item questionnaire assessing exposure to stressful life events (SLEs) throughout the life course. The 10-item PSS is a validated abbreviated version of the full 14-item scale, and is a commonly used tool for assessing subjective appraisal of stress exposure during the previous month [40]. Items are scored on a five-point Likert scale and summed, creating an overall score ranging from 0 to 40, with greater scores indicating greater current perceived stress.

To assess SLE exposure, participants indicated whether they experienced 16 potential SLEs at any point in life, as well as the age (or ages) of occurrence (questionnaire described in greater detail previously [25]). A total lifetime SLE exposure score was created by summing all items, creating a score ranging from 0 to 16, with greater scores reflecting greater SLE exposure. Based on previous evidence suggesting that stressor timing and stressor type may influence the effect of stressor exposure on health outcomes [20], four additional SLE subscales were created: recent SLE exposure (SLEs which occurred ≤ 5 years prior to assessment), non-recent SLE exposure (SLEs which occurred > 5 years prior to assessment), lifetime exposure to acute SLEs (SLEs with a sudden onset and short duration, such as the loss of a close relative), and lifetime exposure to chronic SLEs (SLEs with an extended duration, such as the serious illness of a close relative). If a participant reported the occurrence of any SLE without specifying the age at which it occurred, this SLE was included in the scales reflecting total lifetime SLE, acute SLE, and chronic SLE exposure, but not in the assessment of recent/non-recent SLE exposure. Participants who reported 3+ SLEs without specifying at which age they occurred were removed entirely from all analyses using the recent/non-recent SLE scales.

### AD biomarkers

Levels of three AD biomarkers were assessed from the CSF: Aβ_42_, T-Tau, and P-Tau181. CSF was collected by means of lumbar punctures, which are a standard part of clinical assessments at memory clinics in Sweden. Polypropylene tubes were used, samples were mixed gently to avoid gradient effects, centrifuged for 10 min at 2000×g and subsequently kept at −80 °C until biochemical analysis by means of sandwich ELISA. This process has previously been described in greater detail [56]. T-Tau and P-Tau181 scores displayed significant skewness and were log-transformed, while Aβ_42_ was winsorized at two standard deviations from the mean to reduce the influence of two outlying values. Finally, to improve comparability of the results, all three scores were transformed to have a mean of 0 and a standard deviation of 1.

### Statistical analysis

Sample characteristics and demographics were presented as means and standard deviations for continuous variables and as numbers and percentages for dichotomous variables. Additionally, sex differences in these variables are presented and tested using Mann–Whitney U tests and *χ*^2^ tests. Linear multiple regression models were conducted to assess associations between self-reported stress measures and insomnia. Subsequently, linear multiple regression models were conducted to assess linear interactions between self-reported stress measures and insomnia in relation to AD biomarkers. PSS and insomnia scores were transformed to be centered around their mean, while SLE subscales, which tended to display more skewness, were transformed to be centered around their median. The multiplicative interaction term PSS*insomnia was included to assess interaction between perceived stress and insomnia, while multiplicative interaction terms between SLE subscales and insomnia were included to assess interactions between SLE subscales and insomnia. For the presentation of the results, the insomnia score was additionally transformed to be centered around its 25th and 75th percentile. Associations between self-reported stress measures and AD biomarkers are presented at low and high levels of insomnia, based on the models including the insomnia score centered around its 25th and 75th percentile, respectively.

Since insomnia scores and CSF Aβ_42_ levels appeared to have an inverse U-shaped association in the Co-STAR sample, additional regression models were conducted to assess non-linear interaction between stress measures and insomnia in relation to CSF Aβ_42_ levels. In addition to the centered insomnia score, centered stress variable, and the linear interaction term, these models included the squared insomnia score ((insomnia)^2^) and the interaction term between the relevant stress measure and (insomnia)^2^. Interaction terms (stress variable*insomnia, (insomnia)^2^, and stress variable*(insomnia)^2^) with p-values above 0.05 were removed stepwise to assess the presence of linear and non-linear interactions. All regression models were initially adjusted for age, sex, and years of education (Model 1), followed by additional adjustment for sleep medication use (currently taking/not currently taking) and current depressive symptomatology (Geriatric Depression Scale (GDS)) (Model 2).

Given the known presence of sex differences in the experience of chronic stress and sleep disturbances [61, 66], as well as in the prevalence of AD, sensitivity analyses were conducted after stratification by sex. Using linear multiple regression models, interactions between self-reported stress measures and insomnia in relation to CSF Aβ_42_ levels were assessed separately for men and women, to assess potential sex differences in these interactions.

SPSS Statistics V.27 (IBM, Armonk, New York, USA) and Stata V.16 (StataCorp, College Station, Texas, USA) were used for the analyses. Tests were conducted at a α-level of 0.05. Results are reported using unstandardized coefficients, 95% CIs and p values.

### Ethical considerations

This study involving human participants was approved by the Regional Ethical Review Board (Stockholm) 2014/524-31/1 and conducted in accordance with the Declaration of Helsinki. Participants gave written informed consent to participate in the study before taking part.

## Results

The study sample consisted of 124 participants, with 56 diagnosed with SCI (45.2%) and 68 with MCI (54.8%), as shown in Table 1. Participants were 61.5 (SD = 7.31, range: 47–85) years old on average, and were more likely to be women than men (74 (59.7%) vs 50 (40.3%)). Female participants appeared to be slightly younger than male participants (60.3 vs 63.3), although this difference did not reach statistical significance (*p* = 0.058). Women reported a greater number of depressive symptoms, greater current perceived stress, and a higher total number of SLEs (Table 1). There were no sex differences in insomnia scores, although a greater proportion of women reported current sleep medication use (21.6% vs 10.0%, *p* = 0.091). Mean CSF T-Tau and P-Tau181 levels fell well below common clinical cut-offs for abnormality, while CSF Aβ_42_ levels, which decrease with greater amyloid accumulation in the brain, were well above cut-offs for abnormality on average [49].

**Table 1:** Sample characteristics and demographics

	Full sample		Men		Women		
	*n*		*n*		*n*		*p* (χ^2^/MW U)
Age, m (SD)	124	61.5 (7.31)	50	63.3 (8.20)	74	60.3 (6.44)	0.058
Education (years), m (SD)	124	14.2 (3.25)	50	13.9 (3.13)	74	14.4 (3.33)	0.348
Sleep medication use, n (%)	124	21 (16.9%)	50	5 (10.0%)	74	16 (21.6%)	0.091
Depressive symptoms (__/15), m (SD)	124	5.9 (4.08)	50	4.8 (3.57)	74	6.6 (4.25)	0.013 *
Diagnosis	124		50		74		0.188
- SCI, n (%)		56 (45.2%)		19 (38.0%)		37 (50.0%)	
- MCI, n (%)		68 (54.8%)		31 (62.0%)		37 (50.0%)	
Insomnia score (__/15), m (SD)	124	6.7 (3.52)	50	6.3 (3.13)	74	6.9 (3.75)	0.390
- Difficulty falling asleep (__/5), m (SD)	124	2.1 (1.49)	50	1.8 (1.40)	74	2.2 (1.54)	0.140
- Waking up repeatedly (__/5), m (SD)	124	2.3 (1.47)	50	2.2 (1.36)	74	2.4 (1.54)	0.426
- Early awakening (__/5), m (SD)	124	2.3 (1.44)	50	2.3 (1.26)	74	2.3 (1.56)	0.668
Perceived stress scale (__/40), m (SD)	119	18.4 (7.39)	49	16.8 (7.10)	70	19.5 (7.43)	0.038*
Stressful life events (SLEs)							
- Lifetime SLEs, m (SD)	124	4.4 (2.11)	50	3.7 (1.58)	74	4.9 (2.31)	0.006**
- Recent SLEs (≤ 5 years), m (SD)	115	0.7 (.94)	48	0.5 (.71)	67	0.8 (1.07)	0.463
- Non-recent SLEs (> 5 years), m (SD)	115	3.3 (1.75)	48	2.9 (1.43)	67	3.6 (1.91)	0.075
- Acute SLEs, m (SD)	124	2.2 (1.13)	50	1.9 (1.00)	74	2.5 (1.17)	0.015*
- Chronic SLEs, m (SD)	124	2.2 (1.53)	50	1.8 (1.21)	74	2.4 (1.68)	0.074
Aβ_42_ (ng/L), m (SD)	103	801.7 (210.54)	41	821.9 (212.79)	62	788.5 (209.71)	0.433
T-Tau (ng/L), m (SD)	103	307.4 (152.73)	41	312.9 (161.2)	62	303.7 (148.09)	0.995
P-Tau181 (ng/L), m (SD)	103	44.2 (18.32)	41	45.2 (19.51)	62	43.5 (17.62)	0.853

*Aβ*_*42*_ Beta-amyloid 42; *MCI* mild cognitive impairment; *MW U* Mann–Whitney U test; *P-Tau* Phosphorylated tau; *SCI *subjective cognitive impairment; *SLEs* stressful life events; *T-Tau* total tau

^*^p < 0.05; **p < 0.01

### Associations between self-reported stress and insomnia

Greater current perceived stress was associated with more severe self-reported insomnia after adjusting for both Model 1 covariates and Model 2 covariates (Table 2). No significant associations were found between any of the SLE scales and insomnia symptoms. Interestingly, in models only adjusting for Model 1 covariates, greater exposure to total lifetime SLEs as well as greater exposure to Chronic SLEs appeared to be associated with higher insomnia scores (Total SLEs: b = 0.070, *p* = 0.107; Chronic SLEs: b = 0.099, *p* = 0.097, Table 2). These associations disappeared entirely after further adjustment for Model 2 covariates (Total SLEs: b = − 0.001, *p* = 0.983; Chronic SLEs: b = 0.002, *p* = 0.975), which was due to the addition of depressive symptoms to the models. Any link between lifetime SLE exposure and current insomnia, therefore, appears to be explained by current depressive symptoms.

**Table 2:** Associations between self-reported stress/stressor exposure and insomnia

		Insomnia – Model 1			Insomnia – Model 2		
	*n*	*B*	95% CI	*p*	*B*	95% CI	*p*
Perceived Stress Scale (PSS-10)	119	0.065	0.041—0.089	< 0.001***	0.066	0.032—0.100	<0.001***
Stressful Life Events (SLEs)							
- Total lifetime exposure	124	0.070	− 0.015— 0.155	0.107	−0.001	−0.091—0.090	0.983
- Recent SLEs (≤ 5 years)	115	0.127	− 0.087— 0.340	0.244	0.036	−0.174—0.246	0.735
- Non-recent SLEs (> 5 years)	115	0.037	− 0.067— 0.142	0.483	−0.018	–0.123—0.087	0.732
- Acute SLEs (acute onset, short duration)	124	0.062	− 0.101— 0.224	0.453	−0.006	−0.164—0.151	0.939
- Chronic SLEs (extended duration)	124	0.099	− 0.018— 0.215	0.097	0.002	−0.123—0.127	0.975

Model 1—Adjusted for age, sex, and education; Model 2—Adjusted for Model 1 covariates, sleep medication use, and depressive symptoms (GDS)

^*^ *p* < 0.05; ** *p* < 0.01

### Interactions between self-reported stress and insomnia in relation to AD biomarkers

#### Linear interactions

There was significant interaction between insomnia and perceived stress in relation to CSF Aβ_42_ levels (*b* = − 0.008, *p* = 0.033, Table 3). Greater perceived stress was associated with higher CSF Aβ_42_ levels among participants with low insomnia scores, and with lower CSF Aβ_42_ levels among participants with high insomnia scores. Similarly, insomnia and total lifetime SLEs were found to interact in relation to CSF Aβ_42_ levels (*b* = − 0.032, *p* = 0.003, Table 3), such that greater stressor exposure was associated with higher CSF Aβ_42_ levels among participants with low insomnia scores, and with lower CSF Aβ_42_ levels among participants with high insomnia scores. SLE subscales consistently showed similar patterns, with recent SLE exposure and exposure to chronic SLEs each showing significant interaction with insomnia in relation to CSF Aβ_42_ levels. No interactions were found between stress measures and insomnia in relation to T-Tau or P-Tau181. Analyses adjusting for Model 1 and Model 2 covariates were comparable.

**Table 3:** Interactions between insomnia and chronic stress exposure (perceived stress and stressful life events (SLEs)) in relation to AD biomarkers

	Aβ_42_			T-Tau			P-Tau181		
	*B*	95% CI	*p*	*B*	95% CI	*p*	B	95% CI	*p*
PSS	*n* = 100			*n* = 100			*n* = 100		
Association between PSS & AD biomarker at 25th percentile insomnia score	0.020	−0.030–0.070	0.431	0.031	−0.017–0.078	0.202	0.022	−0.027—0.072	0.370
Association between PSS & AD biomarker at 75th percentile insomnia score	−0.020	−0.065–0.025	0.377	0.035	−0.007—0.078	0.100	0.031	−0.013—0.075	0.167
Interaction coefficient PSS x Insomnia	**−0.008**	**−0.015–−0.001**	0.033*	0.001	−0.006–.008	0.780	0.002	−0.006—0.009	0.636
Lifetime SLE exposure	*n* = 103			*n* = 103			*n* = 103		
Association between SLEs & AD biomarker at 25th percentile insomnia score	0.081	−0.034–0.196	0.168	−0.010	−0.121—0.101	0.859	0.001	−0.114–0.117	0.981
Association between SLEs & AD biomarker at 75th percentile insomnia score	−0.081	−0.194—.032	0.158	−0.049	−0.157—0.060	0.373	−0.049	−0.162–0.064	0.389
Interaction coefficient SLE x Insomnia	**−0.032**	**−0.053—−0.011**	0.003**	−0.008	−0.028—0.012	0.447	−0.010	−0.031–0.011	0.343
Recent SLE exposure (5 years)	*n* = 98			*n* = 98			*n* = 98		
Association between SLEs & AD biomarker at 25th percentile insomnia score	− 0.019	−0.307–0.270	0.898	0.165	−0.117—0.447	0.247	0.254	− 0.041— 0.549	0.091
Association between SLEs & AD biomarker at 75th percentile insomnia score	**– 0.313**	**−0.0562— − 0.065**	0.014 *	0.037	−0.206—0.280	0.764	0.027	− 0.227— 0.282	0.833
Interaction coefficient SLE x Insomnia	**−0.059**	**−0.113—− 0.005**	0.034 *	−0.026	−0.079—0.028	0.340	− 0.045	−.101—.010	0.109
Non-recent SLE exposure	*n* = 98			*n* = 98			*n* = 98		
Association between SLEs and AD biomarker at 25th percentile insomnia score	0.078	− 0.052—.207	0.236	-0.009	-0.133—0.115	0.887	-0.003	-0.135—0.129	0.964
Association between SLEs and AD biomarker at 75th percentile insomnia score	− 0.054	− 0.201— 0.093	0.468	-0.041	-0.182—0.100	0.568	-0.029	-0.178—0.121	0.704
Interaction coefficient SLE x Insomnia	− 0.026	− 0.056— 0.003	0.080	-0.006	-0.035—0.022	0.659	-0.005	-0.035—0.025	0.736
Exposure to acute SLEs	n = 103			*n* = 103			*n* = 103		
Association between SLEs and AD biomarker at 25th percentile insomnia score	− 0.015	− 0.228— 0.198	0.891	0.098	-0.101—0.297	0.329	0.094	-0.114—0.302	0.374
Association between SLEs and AD biomarker at 75th percentile insomnia score	− 0.191	− 0.407—0.026	0.084	0.100	-0.102—0.303	0.326	0.057	-0.155—0.268	0.595
Interaction coefficient SLE x Insomnia	− 0.035	− 0.079— 0.009	0.117	0.000	-0.041—0.042	0.984	-0.007	-0.051—0.036	0.736
Exposure to chronic SLEs	*n* = 103			*n* = 103			*n* = 103		
Association between SLEs and AD biomarker at 25th percentile insomnia score	**0.198**	**0.038—0.358**	0.016 *	-0.070	-0.224—0.083	0.364	-0.043	-0.204—0.118	0.594
Association between SLEs and AD biomarker at 75th percentile insomnia score	− 0.046	− 0.191—0.099	0.531	-0.135	-0.274—0.004	0.057	-0.115	-0.261—0.030	0.120
Interaction coefficient SLE x Insomnia	**− 0.049**	**− 0.078—− 0.019**	0.001 **	-0.013	-0.041—0.015	0.366	-0.014	-0.044—0.015	0.335

Associations between perceived stress and AD biomarkers at low and at high insomnia scores, plus interaction coefficients. Adjusted for Model 2 covariates

Analyses adjusting for age, sex, education, depressive symptoms (GDS), and sleep medication use

Results with *p* <.05 presented in bold

^*^ *p* <.05; ** *p* <.01

#### Non-linear interactions

Following inclusion of (insomnia)^2^ and the interaction term PSS*(insomnia)^2^, PSS no longer showed any interaction with insomnia in relation to CSF Aβ_42_ levels (Table 4). Following stepwise removal of non-significant interaction terms, it appears that the linear interaction between PSS scores and insomnia found previously (Table 3) was explained by the non-linear association between insomnia and CSF Aβ_42_ levels.

**Table 4:** Interactions between insomnia and stress variables in relation to AD biomarkers. Including insomnia-squared and interaction between stressors and insomnia-squared

		Aβ_42_ – Full model with linear interaction			Aβ_42_ – Full model with non-linear interaction			Aβ_42_ – Removing interaction term with highest p-value (if above.05)			Aβ_42_—Removing interaction term with highest remaining p-value (if above.05)		
		*B*	95% CI	*p*	*B*	95% CI	*p*	*B*	95% CI	*p*	*B*	95% CI	*p*
PSS			*n* = 100			*n* = 100			*n* = 100			n = 100	
*Akaike Information Criterion*			*−4.002*			*−2.513*			*−4.511*			*−6.072*	
PSS (mean = 0)		−0.001	−0.05–0.04	0.947	−0.004	−0.05–0.04	0.864	−0.004	−0.05–0.04	0.846	− 0.006	− 0.05–0.04	0.772
Insomnia (mean = 0)		**0.078**	**0.01–.14**	0.017 *	**0.090**	**0.01–0.17**	0.023 *	**0.089**	**0.02–0.15**	0.008 **	**0.090**	**0.03–.16**	0.007 **
Interaction PSS x Insomnia		**−0.008**	**−0.02–0.00**	0.033 *	−0.003	−0.01–0.01	0.545	−0.003	−0.01–0.01	0.531		Removed	
(Insomnia)^2^					−0.012	−0.03–0.00	0.137	−0.012	−0.03–0.00	0.134	**− 0.016**	**− 0.03–0.00**	0.011 *
Interaction PSS x (Insomnia)^2^					0.000	−0.00–0.00	0.969		Removed				
Lifetime SLE exposure			n = 103			n = 103							
*Akaike Information Criterion*			*− 6.930*			*− 15.268*							
SLE (median = 0)		− 0.006	− 0.11–0.10	0.912	−0.101	−0.22–0.02	0.104						
Insomnia (mean 0)		**0.089**	**0.03–0.15**	0.002 **	**0.099**	**0.04–0.15**	<0.001***						
Interaction SLE x Insomnia		**−0.032**	**−0.05– − 0.01**	0.003 **	**−0.036**	**−0.06 –− 0.01**	0.001 **						
(Insomnia)^2^					**−0.014**	**− 0.03–0.00**	0.026 *						
Interaction SLE x (Insomnia)^2^					**0.006**	**0.00–0.01**	0.006 **						
Recent SLE exposure (5 years)			*n* = 98			*n* = 98			*n* = 98			*n* = 98	
*Akaike Information Criterion*			*−9.276*			*−10.885*			*−10.475*			*− 9.276*	
SLE (median = 0)		−0.176	− 0.41–.06	0.133	− 0.303	−0.61–0.00	0.051	− 0.153	− 0.38–0.08	0.188	− 0.176	− 0.41–0.06	0.133
Insomnia (mean 0)		**0.119**	**0.05–0.19**	<.001 ***	**0.142**	**0.07–0.21**	< 0.001 ***	**0.130**	**0.06–0.20**	< 0.001 ***	**0.119**	**0.05–0.19**	< 0.001 ***
Interaction SLE x Insomnia		**− 0.059**	**− 0.11–− 0.01**	0.034 *	**− 0.075**	**− 0.14– −0.01**	0.017 *	− 0.054	− 0.11–0.00	0.052	**− 0.059**	**− 0.11– − 0.01**	0.034 *
(Insomnia)^2^					**− 0.017**	**− 0.03–0.00**	0.026 *	− 0.010	− 0.02–0.00	0.091		Removed	
Interaction SLE x (Insomnia)^2^					0.010	0.00–0.02	0.145		Removed				
Non-recent SLE exposure			*n* = 98			*n* = 98							
*Akaike Information Criterion*			*− 4.547*			*− 15.581*							
SLE (median = 0)	0.008		− 0.11–0.13	0.899	− 0.112	− 0.25–0.02	0.101						
Insomnia (mean = 0)	**0.084**		**0.03–0.14**	0.006 **	**0.097**	**0.04–0.15**	0.001 **						
Interaction SLE x Insomnia	− 0.026		− 0.06–0.00	0.080	− 0.027	− 0.06–0.00	0.061						
(Insomnia)^2^					**− 0.016**	**− 0.03–0.00**	0.009 **						
Interaction SLE x (Insomnia)^2^					**0.009**	**0.00–0.01**	0.002 **						
Exposure to acute SLEs			*n* = 103			*n* = 103							
*Akaike Information Criterion*			*− 1.189*			*− 7.198*							
SLE (median = 0)	−0.109		− 0.29–0.08	0.245	**–0.230**	**−0.46– −0.01**	0.045 *						
Insomnia (mean 0)	**0.094**		**0.03–0.15**	0.003 **	**0.110**	**0.05–0.17**	< 0.001 ***						
Interaction SLE x Insomnia	− 0.035		− 0.08–0.01	0.117	−0.035	− 0.08–0.01	0.116						
(Insomnia)^2^					**− 0.019**	**− 0.03–− 0.01**	0.006 **						
Interaction SLE x (Insomnia)^2^					**0.009**	**0.00–0.02**	0.032*						
Exposure to chronic SLEs			*n* = 103			*n* = 103							
*Akaike Information Criterion*			*− 9.087*			*−18.494*							
SLE (median = 0)	0.068		− 0.07–0.20	0.316	− 0.069	− 0.23–0.09	0.385						
Insomnia (mean 0)	**0.092**		**0.04–0.15**	0.002 **	**0.101**	**0.05–0.16**	< 0.001***						
Interaction SLE x Insomnia	**− 0.049**		**− 0.08 – − 0.02**	0.001 **	**− 0.061**	**– 0.09 – −0.03**	< 0.001***						
(Insomnia)^2^					**− 0.013**	**− 0.03–0.00**	0.025*						
Interaction SLE x (Insomnia)^2^					**0.009**	**0.00–0.02**	0.004**						

Adjusted for Model 2 covariates. Removing the interaction terms with the highest p− value (if > 0.05) step-by-step towards the right (if the SLE*insomnia-squared interaction term is significant, the SLE*insomnia interaction is also kept)

Results with *p* <.05 presented in **bold**

Analyses adjusting for age, sex, education, depressive symptoms (GDS), and sleep medication use; *p < 0.05; **p < 0.01

**Fig. 1:**
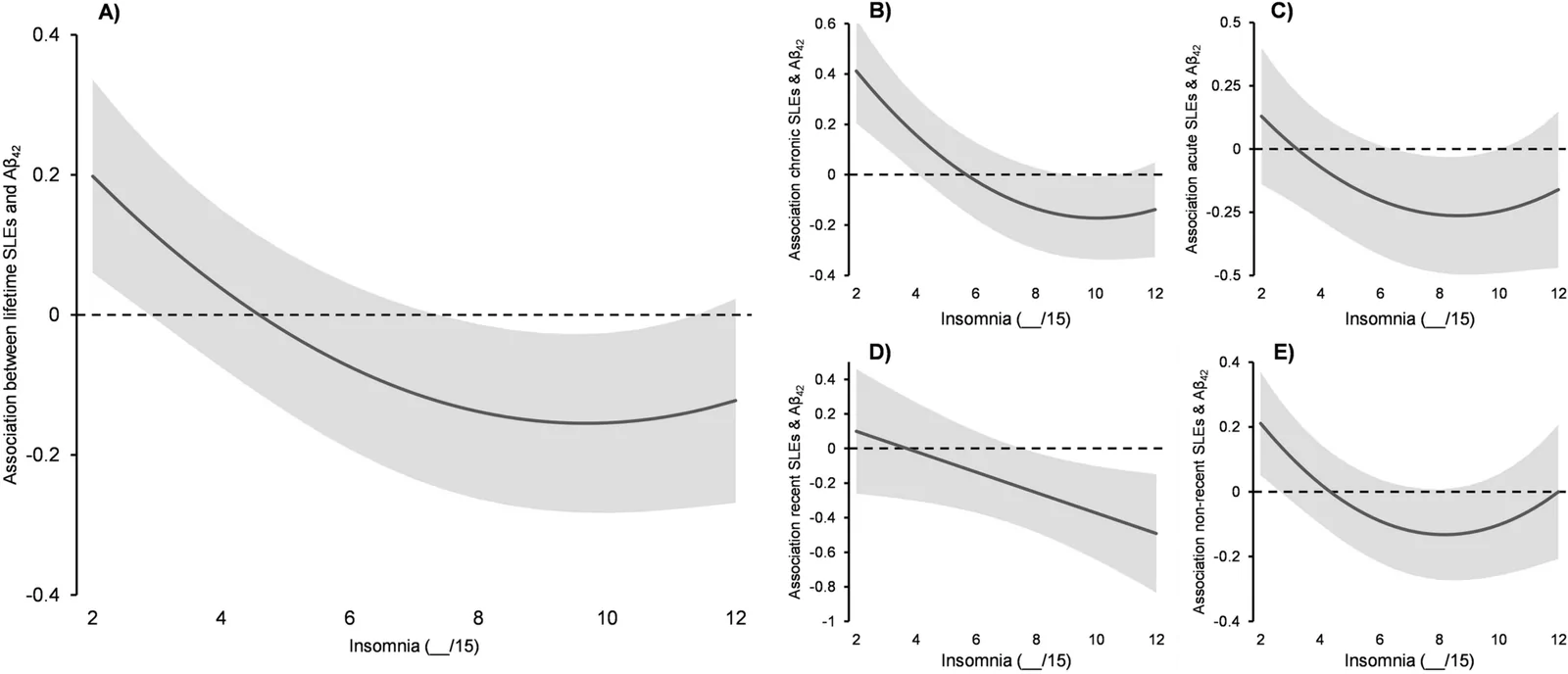
**(**A-E) Associations (β-coefficients) between stressful life event (SLE) exposure and cerebrospinal fluid (CSF) levels of Aβ_42_ across insomnia scores. Results presented for significant SLE*insomnia interactions. Adjusted for Model 2 covariates (age, sex, education, sleep medication use, and depressive symptoms). **1A** Lifetime SLEs and CSF Aβ_42_ across insomnia scores. **1B** Chronic SLEs and CSF Aβ_42_ across insomnia scores. **1C** Acute SLEs and CSF Aβ_42_ across insomnia scores. **1D** Recent SLEs and CSF Aβ_42_ across insomnia scores. **1E** Non-recent SLEs and CSF Aβ_42_ across insomnia scores

Marginal effect plots showing associations between SLE subscales and CSF Aβ_42_ levels across insomnia scores are presented in Fig. 1. Total lifetime SLE exposure, non-recent SLE exposure, exposure to acute SLEs, and exposure to chronic SLEs each showed non-linear interaction with insomnia scores in relation to CSF Aβ_42_ levels (Table 4**, **Fig. 1A–C, E), such that greater SLE exposure was associated with increased CSF Aβ_42_ levels in those with low insomnia scores and with decreased CSF Aβ_42_ levels in those with average-to-high insomnia scores, with this association disappearing in those with very high insomnia scores. Recent SLE exposure did not show non-linear interaction with insomnia, but the linear interaction between recent SLE exposure and insomnia remained after stepwise removal of non-significant interaction terms (Fig. 1D).

### Sex-stratified interactions

Sensitivity analyses were conducted to assess sex-stratified interactions between chronic stress measures and insomnia symptoms in relation to CSF Aβ_42_ levels. Stratified results show fewer significant interactions, given the lower statistical power. However, the direction of the interactions is consistent between men and women, such that the coefficients for the associations between chronic stress measures and CSF Aβ_42_ levels are more negative in those with high insomnia scores compared to those with low insomnia scores (see Supplementary Table 1). Interestingly, the direction of the overall association between SLE subscales and CSF Aβ_42_ levels appears to differ between men and women, such that greater SLE exposure tends to be associated with higher CSF Aβ_42_ levels in men, while greater SLE exposure tends to be associated with lower CSF Aβ_42_ levels in women. Findings should be interpreted with caution, due to the limited sample sizes of the stratified samples.

## Discussion

In this study among patients from a Swedish memory clinic, exposure to SLEs interacted with insomnia in relation to CSF Aβ_42_ levels, suggesting that stressful life events are only associated with amyloid accumulation in the presence of insomnia. The PSS similarly appeared to interact with insomnia, although this interaction disappeared after inclusion of the squared insomnia term. Conversely, no interactions were found between stress exposure and insomnia in relation to T-Tau or P-Tau181. A greater PSS score, but not greater exposure to SLEs, was associated with more severe insomnia.

In line with expectations, greater current perceived stress levels were associated with more severe insomnia symptoms, reflecting the close relationship between stress and sleep disturbances [31]. Similar results have previously been found during the COVID-19 pandemic, when among older adults, greater current chronic stress was found to be associated with insomnia symptoms similar to those used in the present study [31]. The association between exposure to SLEs and insomnia appears to be somewhat more complex. Greater SLE exposure, particularly total lifetime exposure and exposure to chronic SLEs, appeared to be associated with somewhat greater insomnia symptoms after adjustment for age, sex, and education, although these associations did not reach the level of significance. However, these associations disappeared entirely after additional adjustment for depressive symptoms, possibly reflecting a mediating role for depressive symptoms in the association between SLE exposure and insomnia. Stressor exposure has been found to predict depressive symptoms in middle-aged and older adults [4, 26], while a large body of research has found evidence for a close bi-directional relationship between depression and sleep disturbances, including among older adults [8, 55]. SLE exposure throughout the life course may, therefore, increase depressive symptoms, which in turn elevate the risk of insomnia in late life.

SLE exposure was found to interact with insomnia in relation to CSF Aβ_42_ levels. Broadly, greater stressor exposure was associated with lower Aβ_42_ levels, indicative of greater AD pathology, only in the presence of moderate-to-high insomnia scores. Interactions between stress exposure and sleep disturbances in relation to AD pathological processes have not been studied previously, and the mechanisms underlying these interactions, therefore, remain not fully understood. Based on current knowledge, several potential pathways emerge.

Aβ is produced through cleavage of the amyloid precursor protein (APP) [6]. APP cleavage can occur through two distinct pathways: the normal, non-amyloidogenic pathway, which leads to the production of non-toxic fragments, and the abnormal, amyloidogenic pathway, which leads to the production of Aβ [6]. Extensive evidence from animal research suggests that chronic stress-related dysregulation of glucocorticoid hormones shifts APP cleavage towards the amyloidogenic pathway, thereby contributing to greater Aβ production [12]. Meanwhile, sleep promotes the activity of the glymphatic system, which plays a central role in Aβ clearance, and insomnia has consequently been linked to poorer clearance of Aβ [5, 50]. The increased production and decreased clearance resulting from the combined presence of chronic stress and insomnia may, therefore, increase the availability of Aβ beyond the individual effect of each risk factor.

Aβ has a bi-directional association with neuroinflammation, whereby increased levels of Aβ trigger an inflammatory response, which in turn triggers both the further production of Aβ as well as the accumulation of Aβ into plaques [32]. The formation of amyloid plaques, resulting in reduced CSF Aβ levels, therefore depends on neuroinflammation. Chronic stress, and particularly elevated levels of the stress hormone cortisol, are known to be closely related to immune system functioning [2]. While cortisol has anti-inflammatory properties under basal conditions, chronic stress-related dysregulation of cortisol levels has been found to be associated with the release of pro-inflammatory markers [47]. Interestingly, greater exposure to SLEs in childhood or midlife has similarly been found to be associated with neuroinflammation markers, although mostly among those with a history of psychiatric disorders [45]. Meanwhile, in animal research, chronic sleep deprivation has been found to increase the expression of neuroinflammatory markers, which in turn predicted the accumulation of amyloid plaques [33]. Stress- and insomnia-induced neuroinflammation may provide an environment conducive to the accumulation of amyloid plaques, underlying the reduced CSF Aβ_42_ levels seen in participants reporting both greater SLE exposure and insomnia symptoms.

Mohlenhoff et al*.*[37], reviewing relationships between posttraumatic stress disorder, sleep disturbances, and dementia risk, suggest an additional pathway through which insomnia and chronic stress may interact. Insomnia has been found to be associated with reduced hippocampal volume [37, 48]. The hippocampus plays a central role in the regulation of the hypothalamus–pituitary–adrenal (HPA-) axis, activation of which is an integral part of the physiological stress response [53]. Insomnia may, therefore, leave the hippocampus vulnerable to chronic stress-related dysregulation of the HPA-axis [37], and the potential amyloidogenic effects associated with this dysregulation [23]. These proposed potential pathways tend to involve dysregulation of the physiological stress response, which results from prolonged or repeated stress exposure [29]. This may explain why insomnia was found to interact with SLE exposure across the life course, rather than with current perceived stress, which is less reflective of prolonged stress exposure.

No interactions were found in relation to T-Tau or P-Tau181. Since interactions between insomnia and chronic stress measures in relation to AD biomarkers have remained unexplored, the reason for this disparity is unclear. However, differences in the way in which insomnia symptoms correlate with CSF Aβ_42_ levels versus CSF T-Tau or P-Tau levels may play a role [39]. Greater insomnia symptoms were associated with greater CSF Aβ_42_ levels in the present sample, but were not associated with CSF T-Tau or P-Tau181 levels. This is in line with previous research, as numerous studies have found insomnia symptoms, short sleep duration, or poor sleep quality to be associated with elevated Aβ_42_ levels (CSF or plasma), but not with measures of tau in cognitively healthy middle-aged and older adults [14–16, 34, 39]. As amyloid accumulation occurs earlier in disease progression than tau accumulation, it has been suggested that this may indicate that the potential effects of sleep disturbances occur early in the development of AD [14, 16, 39]. Evidence suggests chronic stress exposure may stimulate both the accumulation of Aβ into plaques and the accumulation of tau into neurofibrillary tangles [23, 54]. While the association between chronic stress exposure and Aβ accumulation appears to be affected by insomnia-related elevations in amyloid levels, the lack of association between insomnia symptoms and CSF T-Tau or P-Tau181 levels means that the association between chronic stress exposure and tau accumulation is stable across insomnia scores, leading to the observed lack of interaction in relation to tau.

Additionally, the differing dynamic of Aβ_42_ and tau in CSF may have contributed to the lack of interaction seen in relation to tau measures. While reduced clearance of Aβ may initially lead to an increase in CSF Aβ_42_ levels, greater accumulation of amyloid into plaques leads to a reduction in CSF Aβ_42_ levels [11]. This could contribute to the observed interactions, whereby mild exposure to an individual modifiable risk factors, such as insomnia, is linked to increased CSF Aβ_42_ levels, while greater exposure to multiple interacting risk factors stimulates amyloid accumulation, resulting in reduced CSF Aβ_42_ levels. Conversely, both reduced clearance of tau proteins and more severe pathological changes in the brain (hyperphosphorylation of tau for P-Tau, neurodegeneration for T-Tau) result in elevated CSF tau levels [11]. Therefore, even if insomnia and chronic stress exposure interact in relation to CSF T-Tau and P-Tau levels, this interaction would likely be more subtle than those seen in relation to CSF Aβ_42_ levels. The current sample may have been insufficiently powered to detect these more limited interaction effects.

Given the established sex differences in the occurrence and experience of stress exposure and sleep disturbances, interaction models in relation to CSF Aβ_42_ levels were re-conducted following stratification by sex. The direction of the interaction between self-reported stress measures and insomnia symptoms in relation to CSF Aβ_42_ levels is consistent between men and women. While some interactions found in the overall sample appear to be stronger in men and others appear to be stronger in women, no consistent patterns of sex differences emerged. Notably, greater SLE exposure appears to be associated with reduced CSF Aβ_42_ levels in women, while greater SLE exposure appears to be associated with greater CSF Aβ_42_ levels in men. Animal research suggests that sex differences in the physiological response to stress may underlie these apparent differences [61]. This is supported by research in humans finding that elevated levels of the stress hormone cortisol were associated with greater amyloid accumulation exclusively in women, and specifically in post-menopausal women, highlighting the complex interplay between sex hormones and the physiological stress response [38, 51].

The current study has several notable strengths and limitations. It was one of the first to investigate interactions between two potentially important modifiable AD risk factors in relation to AD pathological changes. The use of a well-characterized cohort of memory clinic patients allowed for the assessment of AD biomarkers from the CSF, providing direct insight into pathological changes which occur in the early stages of disease development. Current stress and insomnia are common occurrences in memory clinic patients, making it a relevant population for the assessment of their interactive effects [13, 30]. However, due to their frequent occurrence, and due to an elevated prevalence of AD pathology in memory clinic patients compared to healthy older adult populations, the generalizability of this study’s findings should be explored in further research.

This study indicates that stress and insomnia symptoms may interact to intensify their respective effects, emphasizing the need to examine combined effects beyond those of individual risk factors. Stress assessments in Co-STAR were comprehensive, which provided the possibility to explore subjective perceived stress as well as the more objective occurrence of SLEs, and to stratify exposure according to stressor timing and stressor type. Conversely, this study also had several limitations. Analyses were cross-sectional, and were, therefore, at risk of reverse causation, whereby stress and sleep disturbances may be the result, rather than the cause, of AD pathological changes. This is of particular concern in the present sample of memory clinic patients, who may already display more advanced pathology. Longitudinal research is needed to further explore these interactions, with assessments starting prior to the development of cognitive decline. The current sample size was insufficient to assess whether sex differences in the central interactions in this study were statistically significant. Sex differences in AD risk, insomnia risk, and the experience of stress and stress-related disorders are well established [7, 36, 44], and future research into these interactions should further explore whether they differ between sexes. Chronic stress and insomnia were assessed by means of self-report, and may have been subject to recall bias. The addition of objective stress measures (e.g., diurnal cortisol patterns or allostatic load composite scores) and objective sleep measures (e.g., actigraphy or a clinical insomnia diagnosis) in future research is needed to fully understand the interplay between these two risk factors. However, care should be taken when designing studies on these interactions using biological stress measures. Common measures, such as cortisol, are affected by various physiological processes beyond chronic stress exposure, including sleep disturbances [1]. Insomnia symptoms specifically have been found to be associated with an altered diurnal cortisol pattern [1]. This could be a concern when studying interactions between insomnia and biological chronic stress measures, as these chronic stress measures may themselves reflect insomnia-related changes. Due to the inflated risk of Type 1 errors resulting from multiple comparisons, examining interactions using both self-reported and biological stress measures was beyond the scope of the present study. Future research could use clinical insomnia diagnoses rather than self-reported insomnia symptoms to assess more long-term sleep disturbances potentially less affected by recall bias. However, sleep disorders often go underdiagnosed among older adults [9], which could bias any findings of such research, if appropriate efforts are not made to reduce this underdiagnosis. Lastly, while AD biomarkers provide a good starting point, further research should explore clinical outcomes, such as brain structure or cognitive function, which may provide insight into the relevance of these interactions in relation to disease symptomatology.

## Conclusions

In conclusion, in memory clinic patients, insomnia symptoms interacted with lifetime stressor exposure in relation to CSF Aβ_42_ levels, such that greater stressor exposure was linked to lower CSF Aβ_42_ levels among those with moderate to high insomnia symptoms. No such interactions were observed in relation to T-Tau or P-Tau181. Current perceived stress was associated with greater insomnia symptoms, whereas lifetime stressor exposure was not. These findings underscore the need to investigate interactive effects of multiple modifiable risk factors in AD development.

## Supplementary Information

Below is the link to the electronic supplementary material.

Supplementary file1 (DOCX 23 KB)

## Data Availability

The datasets used and/or analyzed during the current study are available from the Co-STAR steering committee on reasonable request.

## Abbreviations

Aβ: Beta-amyloid
AD: Alzheimer’s disease
APP: Amyloid precursor protein
CSF: Cerebrospinal fluid
GDS: Geriatric Depression Scale
HPA-axis: Hypothalamus–pituitary–adrenal axis
ICD-10: International classification of diseases 10th revision
KSQ: Karolinska Sleep Questionnaire
MCI: Mild cognitive impairment
PSS: Perceived stress scale
P-Tau: Phosphorylated tau
SCI: Subjective cognitive impairment
SLE: Stressful life event
T-Tau: Total tau
