# Supplementary material for: Insomnia symptoms and stress exposure interact in relation to Alzheimer’s disease biomarkers
Source: J Neurol. 2026 Sep 3;273(10):560. doi: 10.1007/s00415-026-14102-7 (PMC13541817; doi:10.1007/s00415-026-14102-7)
Supplement: Supplementary file 1 [file 415_2026_14102_MOESM1_ESM.docx]

| **Supplementary Table 1.** Sex-stratified **i**nteractions between Interactions between insomnia and chronic stress exposure (perceived stress and stressful life events (SLEs)) in relation to Cerebrospinal fluid beta-amyloid42 (Aβ_42_) levels. Associations between perceived stress and AD biomarkers at low and at high insomnia scores, plus interaction coefficients. | | | | | | | | | |
| --- | --- | --- | --- | --- | --- | --- | --- | --- | --- |
|  | **Full sample** | | |  | **Men** |  |  | **Women** |  |
|  | **Aβ_42_** | | |  | **Aβ_42_** |  |  | **Aβ_42_** |  |
|  | **B** | **95% CI** | **p** | **B** | **95% CI** | **p** | **B** | **95% CI** | **p** |
| **PSS** |  | n = 100 |  |  | n = 40 |  |  | n = 60 |  |
| Association between PSS & AD biomarker at 25^th^ percentile insomnia score | .020 | -.03 - .07 | .431 | .050 | -.04 - .14 | .269 | -.015 | -.08 - .05 | .654 |
| Association between PSS & AD biomarker at 75^th^ percentile insomnia score | -.020 | -.07 - .03 | .377 | -.003 | -.09 - .08 | .950 | -.036 | -.09 - .02 | .216 |
| Interaction PSS x Insomnia | **-.008** | **-.02 - .00** | **.033 *** | -.011 | -.02 - .00 | .092 | -.004 | -.01 - .01 | .424 |
| **Lifetime SLE exposure** |  | n = 103 |  |  | n = 41 |  |  | n = 62 |  |
| Association between SLEs & AD biomarker at 25^th^ percentile insomnia score | .081 | -.03 - .20 | .168 | **.221** | **.04 - .41** | **.020 *** | -.020 | -.18 - .14 | .798 |
| Association between SLEs & AD biomarker at 75^th^ percentile insomnia score | -.081 | -.19 - .03 | .158 | .093 | -.23 - .42 | .564 | **-.141** | **-.27 - -.01** | **.035 *** |
| Interaction SLE x Insomnia | **-.032** | **-.05 - -.01** | **.003 **** | -.026 | -.08 - .03 | .353 | -.024 | -.05 - .00 | .081 |
| **Recent SLE exposure (5 years)** |  | n = 98 |  |  | n = 40 |  |  | n = 58 |  |
| Association between SLEs & AD biomarker at 25^th^ percentile insomnia score | -.019 | -.31 - .27 | .898 | .337 | -.17 - .84 | .185 | -.244 | -.60 - .11 | .172 |
| Association between SLEs & AD biomarker at 75^th^ percentile insomnia score | **-.313** | **-.56 - -.07** | **.014 *** | -.545 | -1.11 - .02 | .057 | **-.330** | **-.62 - -.04** | **.026 *** |
| Interaction SLE x Insomnia | **-.059** | **-.11 - -.01** | **.034 *** | **-.176** | **-.30 - -.06** | **.006 **** | -.017 | -.08 - .05 | .579 |
| **Non-recent SLE exposure** |  | n = 98 |  |  | n = 40 |  |  | n = 58 |  |
| Association between SLEs & AD biomarker at 25^th^ percentile insomnia score | .078 | -.05 - .21 | .236 | **.279** | **.08 - .48** | **.009 **** | -.061 | -.23 - .11 | .472 |
| Association between SLEs & AD biomarker at 75^th^ percentile insomnia score | -.054 | -.20 - .09 | .468 | .191 | -.18 - .57 | .307 | -.130 | -.30 - .04 | .120 |
| Interaction SLE x Insomnia | -.026 | -.06 - .00 | .080 | -.018 | -.09 - .05 | .619 | -.014 | -.05 - .02 | .449 |
| Stratified analyses adjusting for age, education, depressive symptoms (GDS), and sleep medication use; Analyses in full sample additionally adjusting for sex; * p < .05; ** p < .01 | | | | | | | | | |

| **Supplementary Table 1 (cont.)** |  |  |  |  |  |  |  |  |  |
| --- | --- | --- | --- | --- | --- | --- | --- | --- | --- |
|  |  | **Full sample** |  |  | **Men** |  |  | **Women** |  |
|  |  | **Aβ_42_** |  |  | **Aβ_42_** |  |  | **Aβ_42_** |  |
|  | **B** | **95% CI** | **p** | **B** | **95% CI** | **p** | **B** | **95% CI** | **p** |
| **Exposure to acute SLEs** |  | n = 103 |  |  | n = 41 |  |  | n = 62 |  |
| Association between SLEs & AD biomarker at 25^th^ percentile insomnia score | -.015 | -.23 - .20 | .891 | .130 | -.19 - .45 | .417 | -.209 | -.50 - .09 | .162 |
| Association between SLEs & AD biomarker at 75^th^ percentile insomnia score | -.191 | -.41 - .03 | .084 | -.348 | -.80 - .10 | .123 | -.161 | -.42 - .10 | .212 |
| Interaction SLE x Insomnia | -.035 | -.08 - .01 | .117 | **-.095** | **-.18 - -.02** | **.021 *** | .010 | -.05 - .07 | .751 |
| **Exposure to chronic SLEs** |  | n = 103 |  |  | n = 41 |  |  | n = 62 |  |
| Association between SLEs & AD biomarker at 25^th^ percentile insomnia score | **.198** | **.04 - .36** | **.016 *** | **.418** | **.15 - .69** | **.003 **** | .082 | -.12 - .28 | .416 |
| Association between SLEs & AD biomarker at 75^th^ percentile insomnia score | -.046 | -.19 - .10 | .531 | **.334** | **.02 - .65** | **.040 *** | -.170 | -.34 - .00 | .051 |
| Interaction SLE x Insomnia | **-.049** | **-.08 - -.02** | **.001 **** | -.017 | -.08 - .05 | .604 | **-.051** | **-.09 - -.02** | **.006 **** |
| Stratified analyses adjusting for age, education, depressive symptoms (GDS), and sleep medication use; Analyses in full sample additionally adjusting for sex; * p < .05; ** p < .01 | | | | | | | | | |
